# Assessment of the Protection of Dopaminergic Neurons by an α7 Nicotinic Receptor Agonist, PHA 543613 Using [^18^F]LBT-999 in a Parkinson’s Disease Rat Model

**DOI:** 10.3389/fmed.2015.00061

**Published:** 2015-09-02

**Authors:** Sophie Sérrière, Aurélie Doméné, Johnny Vercouillie, Céline Mothes, Sylvie Bodard, Nuno Rodrigues, Denis Guilloteau, Sylvain Routier, Guylène Page, Sylvie Chalon

**Affiliations:** ^1^UMR INSERM U930, Université François Rabelais, Tours, France; ^2^Laboratoires Cyclopharma, Tours, France; ^3^UMR CNRS 7311, Institut de Chimie Organique et Analytique, Université d’Orléans, Orléans, France; ^4^CHRU de Tours, Hopital Bretonneau, Tours, France; ^5^EA3808 – CiMoTheMA, Université de Poitiers, Poitiers, France

**Keywords:** autoradiography, dopamine transporter, 6-hydroxydopamine, neuroinflammation, PET, TSPO

## Abstract

The inverse association between nicotine intake and Parkinson’s disease (PD) is well established and suggests that this molecule could be neuroprotective through anti-inflammatory action mediated by nicotinic receptors, including the α7-subtype (α7R). The objective of this study was to evaluate the effects of an agonist of α7R, PHA 543613, on striatal dopaminergic neurodegeneration and neuroinflammation in a rat model of PD induced by 6-hydroxydopamine (6-OHDA) lesion. Adult male Wistar rats were lesioned in the right striatum and assigned to either the PHA group (*n* = 7) or the Sham group (*n* = 5). PHA 543613 hydrochloride at the concentration of 6 mg/kg (PHA group) or vehicle (Sham group) was intra-peritoneally injected 2 h before 6-OHDA lesioning and then at days 2, 4, and 6 post-lesion. Positron emission tomography (PET) imaging was performed at 7 days post-lesion using [^18^F]LBT-999 to quantify the striatal dopamine transporter (DAT). After PET imaging, neuroinflammation was evaluated in same animals *in vitro* through the measurement of the microglial activation marker 18 kDa translocator protein (TSPO) by quantitative autoradiography with [^3^H]PK-11195. The DAT density reflecting the integrity of dopaminergic neurons was significantly decreased while the intensity of neuroinflammation measured by TSPO density was significantly increased in the lesioned compared to intact striatum in both groups. However, these both modifications were partially reversed in the PHA group compared to Sham. In addition, a significant positive correlation between the degree of lesion and the intensity of neuroinflammation was evidenced. These findings indicate that PHA 543613 exerts neuroprotective effects on the striatal dopaminergic neurons associated with a reduction in microglial activation in this model of PD. This reinforces the hypothesis that an α7R agonist could provide beneficial effects for the treatment of PD.

## Introduction

Parkinson’s disease (PD) is the second most common age-related neurodegenerative disorder after Alzheimer’s disease. It is characterized by the relatively selective death of dopaminergic neurons in the substantia nigra pars compacta leading to a striatal dopamine deficit (1). PD is mainly sporadic and is usually accompanied by motor symptoms, such as bradykinesia, rigidity, postural instability, and resting tremor, although a rising occurrence of non-motor symptoms is now recognized (2). Currently, the treatment of PD is only symptomatic and no efficient neuroprotective or disease modifying approaches are available. The search for such curative treatments requires to explore various molecular pathways involved in order to focus on drugs able to block or curb disease progression. One neuropathological feature of PD is the occurrence of neuroinflammatory processes, which manifests in part through the activation of microglial cell and astrocytes in the substantia nigra (3–5). Regulating neuroinflammation appears therefore to be a potential therapeutic approach; however, despite promising results obtained with different anti-inflammatory drugs in animal models (6, 7), clinical studies have been disappointing (8).

As previously described in the peripheral nervous system (9), it has been shown that downregulation of the microglial activation can be induced through the activation of α7 nicotinic receptors (α7Rs) harbored by microglial cells (10). α7Rs are the acetylcholine nicotinic receptors most represented, with the α4β2 subtype, in mammalian brain and have several pharmacological characteristics, such as a high permeability to calcium, low sensitivity to acetylcholine, and high affinity for α-bungarotoxin (11). They are localized both on neurons (12) and glial cells (13). Besides their effects on neurotransmission, α7Rs are involved in the modulation of neuroinflammatory processes, and agonists of these receptors are more efficient than acetylcholine at inhibiting the inflammatory signaling and production of pro-inflammatory cytokines from immune cells (14). In addition, several studies conducted in animal models support the idea that drugs acting at nicotinic acetylcholine receptors may be beneficial for PD (15). In particular, it has recently been suggested that the beneficial effect on the dopaminergic function observed with the α7R agonist ABT-107 could be linked to a reduction of the glutamate excitotoxicity leading to the promotion of neuronal integrity (16).

In this study, we used a selective agonist of α7R, PHA 543613 (17, 18), in a rat model of PD induced by unilateral striatal administration of 6-hydroxydopamine (6-OHDA) to investigate whether this compound has a protective effect on dopaminergic neurons through an anti-inflammatory mechanism. For this aim, we measured the striatal dopamine transporter (DAT) by *in vivo* positron emission tomography (PET) imaging using the fluorinated derivative of PE2I, *(E)-N*-(4-fluorobut-2-enyl)2β-carbomethoxy-3β-(4’-tolyl)nortropane or LBT-999 that we previously developed (19, 20). The neuroinflammation was evaluated through the density of the 18 kDa translocator protein (TSPO) using a quantitative autoradiographic method with the reference ligand of TSPO [^3^H]PK-11195, which has been widely used in rodent and human brains (21–24).

## Materials and Methods

### Animals

All procedures were conducted in accordance with the European Community Council Directive 2010/63/EU for laboratory animal care and the experimental protocol was validated by the Regional Ethical Committee (Authorization N°00434.02). Experiments were carried out on adult male Wistar rats (CERJ, France) weighing 290–300 g at the beginning of experiments. Animals were housed in groups of two per cage in a temperature (21 ± 1°C) and humidity (55 ± 5%) controlled environment under a 12-h light/dark cycle, with food and water available *ad libitum*.

### 6-OHDA lesion

The experimental design was performed according to a previously described procedure (25). Twenty minutes before surgery, rats were injected intra-peritoneally with pargyline (50 mg/kg, Sigma, Saint-Quentin Fallavier, France). Rats were anesthetized with isoflurane (4%, 500 mL/min) and placed on a stereotaxic apparatus (Stoelting, Phymep, Paris, France) and maintained under isoflurane 2.5% (500 mL/min) during surgery. The skull was exposed and small holes were made with a dental drill. Lesion was carried out by unilateral intrastriatal injection of 6-OHDA hydrochloride (1 mg/mL, Tocris Bioscience, Bristol, UK). A total of 10 μg of 6-OHDA was administered in two areas of the right striatum (1 mg/mL in 0.01% ascorbic acid, pH 4.5, i.e., 5 μg in 5 μL for each area) with a Hamilton syringe (gage 25, Hamilton, Massy, France) at a flow rate of 1 μL/min. Coordinates from bregma were AP = +0.5 mm, L = −2.5 mm, P = −5 mm, and AP = −0.5 mm, L = −4 mm, P = −5 mm according to Paxinos and Watson atlas (26). The needle was left in place for 4 min after injection and then removed slowly to optimize toxin diffusion. After surgery, the rats were given buprenorphine (0.05 mg/kg sub-cutaneously) for postoperative pain and were allowed to recover from surgery for 7 days before being subjected to the imaging experiments.

### Treatment with the α7 receptor agonist PHA 543613

PHA 543613 hydrochloride (Tocris Bioscience, Bristol, UK; 17, 18) was dissolved in sterile water and intra-peritoneally injected at the concentration of 6 mg/kg (300 μL/300 g body weight) 2 h before 6-OHDA lesioning and then at days 2, 4, and 6 post-lesion (cumulative dose of 24 mg/kg). Twelve rats were included in this study and separated into two groups as follows: seven lesioned rats received the treatment (PHA group) and five lesioned rats received intra-peritoneally injection of vehicle at the same time points (Sham group).

### Preparation of the tracer

No-carrier-added [^18^F]LBT-999 was prepared as previously described (27). The tracer was produced via direct nucleophilic substitution from its chloro analog by adding 3 mg of the precursor in 1 mL of DMSO to the dry [^18^F] KF/K_2.2.2_ complex. After heating at 165°C for 10 min, the mixture was cooled and purified by HPLC (Alltima, C18, 250 × 10 mm, 5 μm column) using ammonium acetate 0.1M/acetonitrile 4/6 as the mobile phase at a 4 mL/min flow rate. In these conditions, time retention was 13.5 min. The desired fraction was collected, diluted in water, and the [^18^F]LBT-999 was trapped on a *t*-C18 light SepPak cartridge. The cartridge was rinsed with 5 mL of injectable water, and the [^18^F]LBT-999 was eluted with 0.5 mL of ethanol. Formulation was completed by the addition of 3.5 mL of NaCl 0.9%. Quality control was performed by HPLC (Alltima, C18, 250 × 4.6 mm, 5 μm column) using ammonium acetate 0.1M/Acetonitrile 3/7. [^18^F]LBT-999 was obtained with a radiochemical purity >98% and with a mean specific activity of 65 ± 10 GBq/μmol.

### PET imaging and data analyses

Positron emission tomography imaging was performed at 7 days post-lesion. Acquisitions were made on a microPET eXplore VISTA-CT system (GE Healthcare, France) which has an effective axial/trans axial field of view (FOV) of 4.8/6.7 cm, a spatial resolution less than 2 mm and a sensitivity above 2.5% in the whole FOV. Animals were anesthetized with isoflurane (Baxter, France), at 4–5% in O_2_ for induction and then 1.5–2% during scanning. For imaging, each rat was placed on a thermo-regulated bed (Minerve, France) in the prone position with a nose cone. The brain was positioned on the center of the FOV. Before PET acquisition, a 5-min computed tomography (CT) scan was acquired for attenuation correction. A bolus injection of 37 MBq/300 g body weight of [^18^F]LBT-999 in saline was administered into the tail vein. During PET acquisition, the respiratory rate and body temperature were monitored and kept as constant as possible. Each acquisition lasted 50 min and PET list-mode scans were rebinned into 27 frames: 4 10-s frames followed by 4 20-s frames, 4 60-s frames, 14 180-s frames, and 1 120-s frame. Each PET scan was corrected for random, scatter, and attenuation, and the images were reconstructed using a 2-D OSEM algorithm (GE Healthcare, France) into voxels of 0.3875 mm × 0.3875 mm × 0.775 mm. All images were analyzed using PMOD (3.403, PMOD Technologies, Zurich, Switzerland, www.pmod.com). The PET-corrected images were used for standard uptake value (SUV) calculations. For each PET scan, data were summed over the first 5 min after radiotracer injection to create a pseudo perfusion image. This image reflects the initial flow-dependent activity and was registered with the CT image through a known hardware registration (PET to CT transformation). CT scans were also recorded using a rat brain magnetic resonance imaging template (MRI-Template) (PMOD) (28) and a rat brain MRI-Template to CT transformation was saved. All PET images, after checking for potential head movements, were co-registered in a single interpolation to the Schiffer rat brain MRI-Template by a combination of these two transformations (MRI-Template to CT, and PET to CT transformations). The inverse combined transformation was calculated. The Schiffer MRI-template was processed in the PET space images using the inverse transformation applied on the originals dynamic PET data and statistics for the regions of interest (ROIs) were extracted. PET images were analyzed with the ROIs for the left striatum (i.e., intact striatum, IST), right striatum (i.e., lesioned striatum, LST), and cerebellum (CE). In this study, the standard uptake value ratio (SUVr) was used as quantitative criterions. All SUVrs were calculated using the CE as the reference region.

### Autoradiographic study

After the PET scan, rats were humanely killed by decapitation under light isoflurane anesthesia, and their brains were carefully removed on ice for autoradiographic experiments according to Ref. (25). Brains were frozen in isopentane cooled at −35°C and stored at −80°C. Coronal brain sections 20-μm thick were cut with a cryostat (CM 3050S, Leica, Germany) at −20°C, collected on gelatinized slides and stored at −80°C for at least 4 days. A total of six sections per brain were studied for each animal. The density of TSPO binding sites was measured by *in vitro* autoradiographic experiments using [^3^H]PK-11195 (specific activity 3.06 GBq/μmol; Perkin Elmer, Norwalk, CT, USA) at 1 nmol/L in a 50-mmol/L Tris–HCl buffer pH 7.4. Brain sections were allowed to equilibrate at room temperature (RT) for 3 h, then were incubated with 1 nmol/L [^3^H]PK-11195 in 50 mmol/L Tris–HCl buffer pH 7.4 at RT for 60 min. Non-specific binding was assessed in the presence of 1 μmol/L PK-11195 (Sigma Aldrich, France). Sections were rinsed twice in ice cold buffer (4°C) for 5 min, then briefly in distilled water at 4°C and dried at RT. Dry sections were made conductive by an application of metal electric tape (3M, Euromedex) on the free side and then placed in the gas chamber of the β-imager™ 2000 (Biospace Lab, Paris, France). Acquisitions were collected for 4 h. Two anatomical ROIs, i.e., the LST and IST were selected manually and identified in Paxinos and Watson atlas (26). Using the β-vision software (Biospace Lab, Paris, France), the level of bound radioactivity was directly determined by counting the number of β-particles emitted from the delineated area. The radioligand signal in the ROIs was measured for each rat and expressed as counts per minute per square millimeter (cpm/mm^2^). Specific binding was determined by subtracting non-specific binding from total binding. Radioactivity was quantified using an image analyzer (M3-vision Biospace Instruments, France). The percentage increase of TSPO binding in LST vs. IST was calculated as:

$$\frac{\left[\text{LST}-\text{IST}\right]}{\text{IST}}*100$$

### Statistical analysis

For PET imaging and autoradiographic studies, results were expressed as mean ± mean standard error (SEM). Comparisons between the binding in the LST and IST were performed using the Wilcoxon test one-tailed. To compare the two groups of rats (PHA vs. Sham), a Mann–Whitney test was used. The level of significance was *p* ≤ 0.05. Correlation between PET imaging and autoradiography was estimated by using a one-tailed Spearman test (GraphPad Instat, GraphPad Software, San Diego, CA, USA). The level of significance was *p* ≤ 0.05. Statistical analyses were performed using the GraphPad Prism software version 5.

## Results

### Animals

No physiological issues and no difference in body weight were observed between animals in the PHA and Sham groups (weight on the day of lesion: 300 ± 4 vs. 297 ± 6 g, respectively; weight at day 7 post-lesion: 313 ± 5 vs. 301 ± 6 g, respectively).

### PET imaging

**Figure 1:**
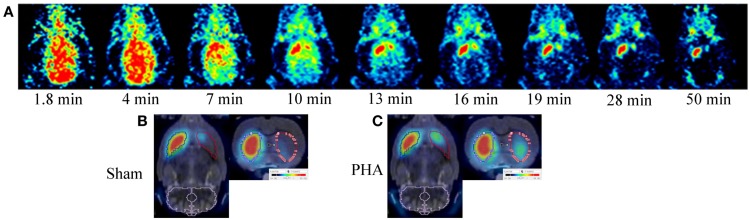
**PET brain images with [^18^F]LBT-999**. **(A)** Coronal PET images in a 6-OHDA lesioned rat with [^18^F]LBT-999 from 1.8 to 50 min after bolus injection of the tracer. PET brain static sagittal and coronal images with [^18^F]LBT-999 co-registered with the MRI-Template in a Sham rat **(B)** and in a PHA rat **(C)**.

Positron emission tomography images are presented in Figure 1. After [^18^F]LBT-999 injection, a progressive accumulation of radioactivity was observed mainly in the IST (Figure 1A). Static PET images revealed that accumulation of [^18^F]LBT-999 on the lesioned striatum was greater in PHA rats than in Sham rats (Figures 1B,C).

**Figure 2:**
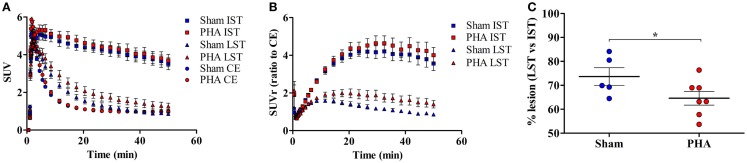
**Quantitative results from PET brain images with [^18^F]LBT-999**. **(A)** Mean-time activity curves of [^18^F]LBT-999 SUVs in the IST, LST, and CE, in Sham (blue) and PHA (red) rats. **(B)** Mean-time activity curves of SUVr to CE of tracer accumulation in IST and LST in Sham (blue) and PHA (red) rats. **(C)** Percentage of lesion in Sham (blue) and PHA (red) rats. *p < 0.05 compared with Sham (Mann–Whitney test). Abbreviations: CE: cerebellum, IST: intact striatum, LST: lesioned striatum, SUV: standard uptake value, SUVr: standard uptake value ratio to CE.

The time activity curves (Figure 2A) showed a rapid uptake of [^18^F]LBT-999 in the ROIs (i.e., the IST, LST, and CE) after intravenous bolus injection. In the CE, the uptake decreased rapidly and remained low and stable after 20 min post-injection in both groups (SUV in the PHA group: 0.97 ± 0.06, and 0.99 ± 0.08 in the Sham group). The uptake remained high and stable in the IST in both groups (SUV in the PHA group: 3.99 ± 0.33, and 3.84 ± 0.31 in the Sham group). By contrast, the values observed in the LST were reduced in comparison to those in the IST in both groups (SUV in the PHA group: 1.41 ± 0.19, and 0.98 ± 0.11 in the Sham group).

The analysis of SUVrs to CE (Figure 2B) showed a significant reduction in the LST compared to the IST, both in the PHA (1.58 ± 0.23 vs. 4.34 ± 0.42, *p* < 0.05 Wilcoxon test) and Sham (0.99 ± 0.07 vs. 3.94 ± 0.37, *p* < 0.05 Wilcoxon test) groups. These SUVrs were similar in the IST in both groups (PHA: 4.34 ± 0.42 and Sham: 3.94 ± 0.37), whereas in the LST the SUVr was slightly higher in the PHA than in the Sham group (1.58 ± 0.23 and 0.99 ± 0.07, respectively). In addition, the percentage of decrease in the SUVr (which reflects the intensity of the lesion) was significantly lower in the PHA group compared to the Sham group (64.6 ± 2.9 vs. 73.7 ± 3.7%, *p* < 0.05, Mann–Whitney test) (Figure 2C).

### Autoradiographic study

**Figure 3:**
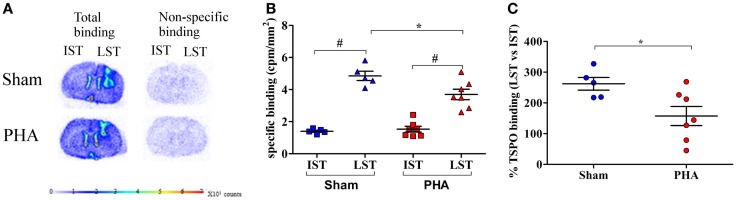
**Autoradiographic analysis of TSPO density with [^3^H]PK-11195**. **(A)** Representative total (left side) and non-specific (right side) binding of [^3^H]PK-11195 obtained on 20 μm-thick coronal brain sections in Sham rats (upper panel) and in PHA rats (lower panel). Note that the cortical signal reflects the binding resulting from the mechanical lesion induced by the needle. **(B)** Quantitative autoradiographic measurements of TSPO density expressed as specific binding of [^3^H]PK-11195 in IST and LST from Sham (blue) and PHA (red) rats (mean cpm/mm^2^ ± SEM). **(C)** Percentage of TSPO binding in LST vs. IST (mean ± SEM%) from Sham (blue) and PHA (red) rats.*p < 0.05 (Mann–Whitney test). ^#^*p* < 0.05 (Wilcoxon test). Abbreviations: TSPO, translocator protein; IST, intact striatum; LST, lesioned striatum.

The TSPO density was evaluated on adjacent brain sections by [^3^H]PK-11195 binding in the IST and LST from each rat in both groups as illustrated in Figure 3A. The specific binding of [^3^H]PK-11195 (Figure 3B) in the IST was low and similar between the PHA and Sham groups (1.53 ± 0.18 and 1.40 ± 0.29 cpm/mm^2^, respectively). In the LST of both groups, this binding was significantly increased (PHA group: 3.69 ± 0.32 vs. 1.53 ± 0.18 cpm/mm^2^ in the IST *p* < 0.05 Wilcoxon test; Sham group: 4.85 ± 0.29 vs. 1.40 ± 0.06 cpm/mm^2^ in the IST, *p* < 0.05 Wilcoxon test). However, this increase was significantly lower in the PHA group than in the Sham group (24% lower, *p* < 0.05, Mann–Whitney test). In addition, as shown in Figure 3C, the percent of increase of TSPO binding in LST vs. IST was significantly lower in the PHA group compared to the Sham group (157.5 ± 30.9 vs. 262.2 ± 20.6%, *p* < 0.05, Mann–Whitney test).

### Correlation between PET imaging and autoradiography

**Figure 4:**
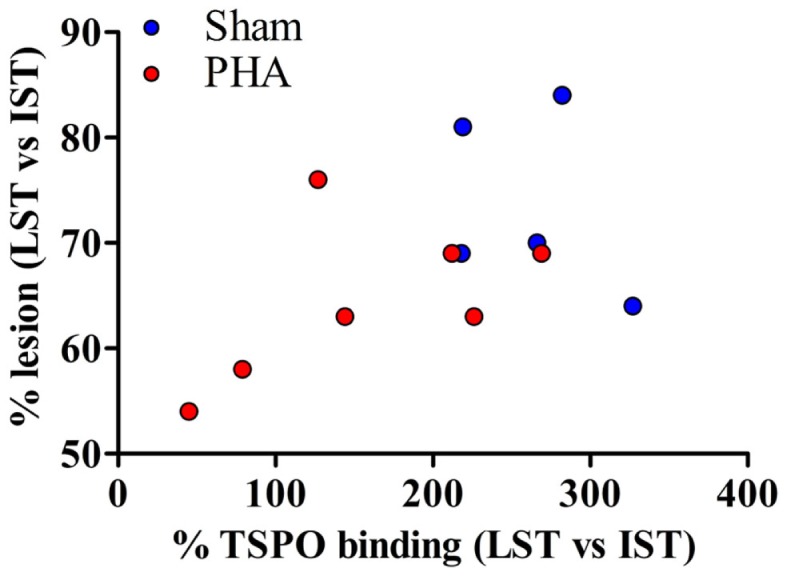
**Correlation between PET imaging and autoradiography**. The correlation is reported for both groups. Sham rats are represented in blue and PHA rats are in red. A one-tailed Spearman test was used (*p* = 0.05, *rho* = 0.49).

The correlation between PET imaging results (expressed as the degree of lesion, i.e., % tracer binding in LST vs. IST) and autoradiography (expressed as a percent of neuroinflammation, i.e., % TSPO binding in LST vs. IST) is shown in Figure 4. The degree of lesion measured by PET imaging was positively correlated with the intensity of neuroinflammation (*p* = 0.05, *rho* = 0.49).

## Discussion

To date, no treatment has been recognized as useful neuroprotective approach for PD. Neuroinflammation is believed to be a key pathophysiological mechanism that could be targeted to achieve neuroprotection (29). A number of epidemiological and experimental evidence have converged to demonstrate a neuroprotective effect of nicotine in PD (30), involving particularly the α7-subtype of nicotinic receptors (31). Interestingly, this effect could be at least partly mediated by the modulation of neuroinflammation affected by these receptors (11). On this basis, we evaluated in this study the effects of a potent agonist of α7R, PHA 543613, on the neurodegeneration of dopaminergic neurons and associated microglial activation in a rat model of PD.

To date, little is known on the potential effects of α7R agonists in PD. As we aimed to induce a potential neuroprotective effect, we used a partial lesion model which corresponds to an early symptomatic stage of PD (32, 33).

Because the striatal DAT has proven to be a marker that correlates with the level of dopaminergic denervation (30, 34), we studied the integrity of striatal dopaminergic nerve endings using PET imaging with the fluorinated derivative of PE2I, *(E)-N*-(4-fluorobut-2-enyl)2β-carbomethoxy-3β-(4’-tolyl)nortropane or LBT-999 that we previously developed (19, 20). We recently demonstrated the ability of [^18^F]LBT-999 to quantify *in vivo* the DAT in the rat brain with high reproducibility, sensitivity, and specificity (27). In addition, this tracer has proven useful in a rat model of PD to quantify the lesions and as a treatment for cell restoration (35).

In order to evaluate the effects of PHA on neuroinflammation, we measured the density of the 18 kDa TSPO in the brain of PD rats after exploration by PET imaging. Indeed, the inflammatory reaction in the brain involves the activation of microglia (36) leading to a dramatic increase in the expression of the TSPO which can thus be considered as a sensitive biomarker of this activation (37, 38). We used a quantitative autoradiographic method with the reference ligand of TSPO [^3^H]PK-11195, which has been widely used in rodent and human brains (21–24).

Our PET experiment showed that at 7 days after 6-OHDA lesion, the specific accumulation of [^18^F]LBT-999 in the striatum, measured as the SUVr to CE as reference region, was high and similar on the intact side of all animals, and reduced on the lesioned side, as expected. However, we observed that the degree of lesion of dopaminergic neurons on the lesioned side, calculated as the percentage of tracer binding in the lesioned vs. intact striatum was slightly but significantly reduced in the PHA-treated group compared to Sham group (64 vs. 74% lesion, respectively). These results are in agreement with a partial neuroprotective effect of the α7R agonist on dopaminergic nerve endings. It is known that PHA 543613 is a potent and selective α7R agonist (17, 18), which has been shown to reduce induced brain edema in a mouse model through the inhibition of GSK-3β (39, 40). Similarly, PHA 568487, an α7R agonist closely related to PHA 543613, has been shown to reduce the severity of an ischemic stroke (41).

In rodent models of PD, neuroprotective effects have already been described with different α7R agonists, i.e., DMXBA (42), PNU-282987 (43) and more recently ABT-107 (16). In several of these studies microglial activation was reduced by treatment with α7R agonists, but the effects on neuroinflammatory parameters were observed at much earlier time’s post-lesion than the effects on dopaminergic parameters (42, 43). We demonstrated herein a strong relationship between the effects of the α7R agonist on the dopaminergic neurons integrity and on neuroinflammation, because α7R agonist treatment reduced both dopaminergic neuron loss and microglial activation in the same animal. This is reinforced by the fact that we showed a significant positive correlation between the decrease of [^18^F]LBT-999 accumulation and [^3^H]PK-11195 binding, which confirms that the upregulation of TSPO is directly correlated with the degree of neuronal damage (38).

Taking into account the small number of animals studied, our present findings can be considered as preliminary. In addition, several complementary experiments such as the evaluation of dopaminergic neuron integrity through TH immunostaining, behavioral tests assessing the dopaminergic function, and different experimental designs with various doses and administration points would be performed to confirm our findings. Nevertheless, we showed that *in vivo* PET exploration in rodent models can be useful to evaluate different neuroprotective approaches using a variety of pharmacological compounds and experimental designs. In this field, the use of the TSPO PET tracer [^18^F]DPA-714 in parallel to PET tracers of neurodegeneration would be of great value in rodent models of neurodegenerative disease, as recently done (44).

Our findings indicate that PHA 543613, a high affinity and selective α7R agonist, partially preserved the integrity of striatal dopaminergic nerve endings and reduced neuroinflammation in 6-OHDA-lesioned rats. This reinforces the hypothesis that α7R agonists may be beneficial in PD.

## Conflict of Interest Statement

The authors declare that the research was conducted in the absence of any commercial or financial relationships that could be construed as a potential conflict of interest.

## Abbreviations

α7R, α7 nicotinic receptor; CE, cerebellum; IST, intact striatum; LST, lesioned striatum; SUV, standard uptake value; SUVr, standard uptake value ratio to CE.
